# Seronegative Intestinal Villous Atrophy: A Diagnostic Challenge

**DOI:** 10.1155/2016/6392028

**Published:** 2016-10-10

**Authors:** Cláudio Martins, Cristina Teixeira, Suzane Ribeiro, Daniel Trabulo, Cláudia Cardoso, João Mangualde, Ricardo Freire, Ana Luísa Alves, Élia Gamito, Isabelle Cremers, Ana Paula Oliveira

**Affiliations:** Department of Gastroenterology, Setúbal Hospital Center, São Bernardo Hospital, Setúbal, Portugal

## Abstract

Celiac disease is the most important cause of intestinal villous atrophy. Seronegative intestinal villous atrophy, including those that are nonresponsive to a gluten-free diet, is a diagnostic challenge. In these cases, before establishing the diagnosis of seronegative celiac disease, alternative etiologies of atrophic enteropathy should be considered. Recently, a new clinical entity responsible for seronegative villous atrophy was described—olmesartan-induced sprue-like enteropathy. Herein, we report two uncommon cases of atrophic enteropathy in patients with arterial hypertension under olmesartan, who presented with severe chronic diarrhea and significant involuntary weight loss. Further investigation revealed intestinal villous atrophy and intraepithelial lymphocytosis. Celiac disease and other causes of villous atrophy were ruled out. Drug-induced enteropathy was suspected and clinical improvement and histologic recovery were verified after olmesartan withdrawal. These cases highlight the importance for clinicians to maintain a high index of suspicion for olmesartan as a precipitant of sprue-like enteropathy.

## 1. Introduction

The combination of diarrhea, weight loss, and villous atrophy (VA) is usually associated with celiac disease (CD) [1]. The diagnosis of CD is supported by positive serological tests, celiac permissive HLA type, and symptomatic and histologic response to a gluten-free diet (GFD). Although CD is the most common cause of VA, patients with VA and negative celiac serology are encountered, placing a diagnostic and therapeutic dilemma. Testing for other conditions such as* Crohn*'s disease, autoimmune enteropathy, microscopic enterocolitis, and infectious enteritis should be warranted. Another possible cause of VA has recently garnered more attention—drug-induced enteropathy. Reports of damage to the intestinal villi by pharmaceuticals have been described previously with the use of immunosuppressive drugs, in particular azathioprine and methotrexate [2, 3]. Recently, a new drug was linked to sprue-like enteropathy—the oral angiotensin-receptor blocker (ARB) olmesartan. To date, the pathophysiologic mechanisms of olmesartan-induced sprue-like enteropathy (OISLE) remain unknown, although a cell-mediated immune response and a genetic predisposition are suggested.

Herein, we report two uncommon cases of severe OISLE and discuss the natural history of this entity.

## 2. Case Report 1

A 53-year-old Caucasian female was admitted to our Department with a 6-month clinical picture of diarrhea and significant involuntary weight loss (20% body weight). She reported between 6 and 10 daily episodes of bulky, watery, and nonbloody diarrhea, abdominal bloating, and, more recently, severe asthenia. She denied abdominal pain, fever or other symptoms suggestive of local or systemic infections, recent travels or sick contacts, prior exposure to health care environments, or changes in her diet habits or medications within the last few years. Apart from arterial hypertension treated with olmesartan 40 mg/daily for the last six years, her past medical and surgical history were unremarkable. The patient did not respond to initial therapeutic approach, including probiotics, an opioid-receptor agonist, and gluten- and lactose-free diet, as well a trial of oral antibiotic for possible small bowel bacterial overgrowth.

On admission, physical examination showed pallor and muscle wasting. Initial blood tests showed mild microcytic anemia (11 g/dL; N: 11.5–15.5), leukopenia (3.8 × 10^9^/L; N: 4.5–10), thrombocytopenia (130 × 10^9^/L; N: 150–400), and hypokalemia (2.8 mmol/L; N: 3.5–5). The rest of the laboratory study was unremarkable including erythrocyte sedimentation rate, C-reactive protein, liver and kidney tests, total protein, albumin, and immunoglobulin levels, B12 vitamin, folic acid, thyroid-stimulating hormone, anti-tissue transglutaminase antibodies, and serology for human immunodeficiency virus (HIV) and cytomegalovirus. Stool culture for infectious agents and polymerase chain reaction testing for* C. difficile *toxins A and B were negative.

**Figure 1:**
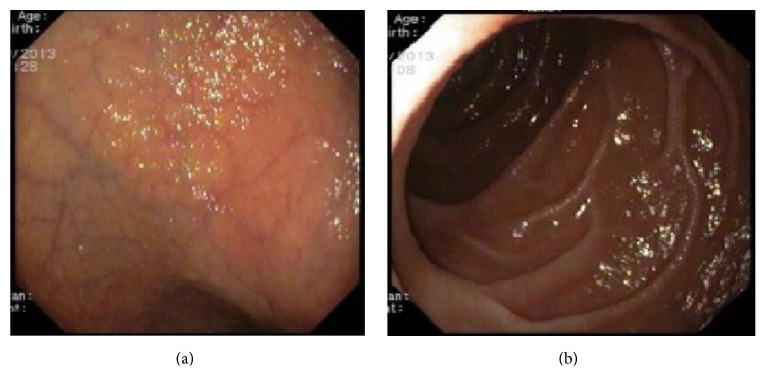
Endoscopic findings. (a) Visible blood vessels on the duodenal bulb. (b) Attenuation of mucosal folds on the second portion of duodenum.

**Figure 2:**
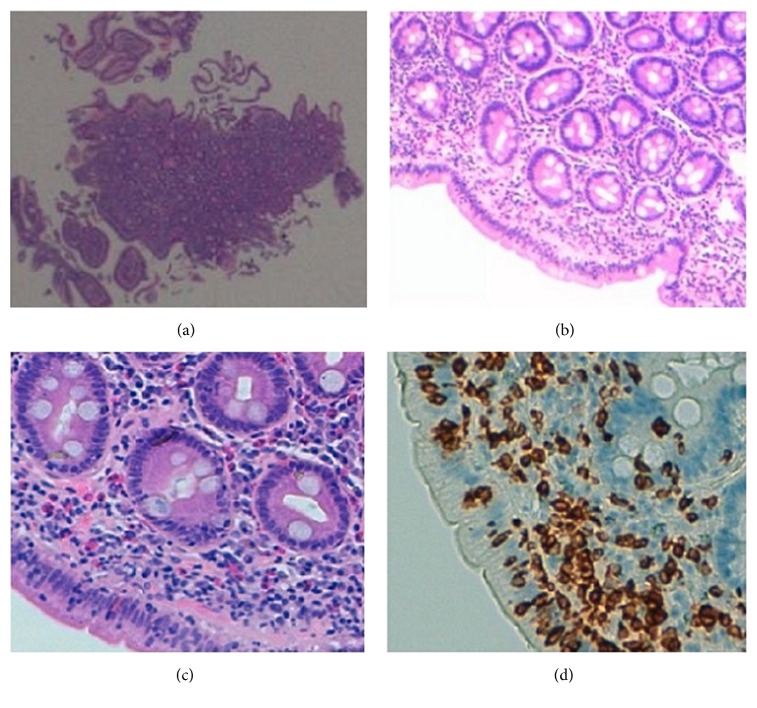
Duodenal histopathological features. (a, b, c) Partial villous atrophy with lymphocytic inflammatory infiltrate in lamina propria and surface intraepithelial lymphocytosis (H&E). (d) CD3 immunostaining shows pathological increase of intraepithelial lymphocytes (IHC ×200).

The subsequent workup included abdominopelvic computed tomography scan which was negative for pancreatic abnormalities or other suspicious lesions for malignancy; total colonoscopy and terminal ileoscopy with random biopsies that were normal and with no evidence of inflammatory bowel disease or microscopic colitis; and upper endoscopy that showed attenuation of duodenal folds (Figure 1) and histopathological examination confirmed a partial villous atrophy and intraepithelial lymphocyte infiltration with no signs of granulomas, microorganisms, or malignancy (Figure 2).

A diagnostic hypothesis of OISLE was placed and, therefore, this drug was withdrawn and switched to lisinopril. Prompt improvement was achieved within 72 h with resolution of diarrhea and normalization of laboratory tests. Upper endoscopy was repeated two months later showing normal duodenal appearance and complete recovery of duodenal villi and no intraepithelial lymphocyte infiltration, on histology. At six-month follow-up, the patient remained asymptomatic with complete recovery of weight loss.

## 3. Case Report 2

A 65-year-old Caucasian female with arterial hypertension under olmesartan 40 mg/daily in the last four years was admitted to our Department with a 2-month clinical picture of watery and nonbloody diarrhea (7–10 stools/day), diffuse abdominal pain, fatigue, anorexia, and involuntary weight loss (10% body weight). She denied fever and any other relevant epidemiologic context. She was previously evaluated by her family doctor who prescribed empiric antibiotic but with no improvement. On admission, physical examination showed dehydration, hypotension, and tachycardia. Laboratory evaluations revealed normocytic anemia (10.9 g/dL; N: 11.5–15), urea 84 mg/dL (N: 21–43), creatinine 3.63 mg/dL (N: 0.6–1.1), serum potassium (2.5 mmol/L; N: 3.5–5), and albumin 3.1 g/dL (N: 3.4–4.8). Serology for celiac disease and thyroid hormone levels and stool analysis for infectious agents (*C. difficile*, virology, bacteriology, mycobacteriology, and parasitology) and serology for HIV, cytomegalovirus, and herpes simplex virus were all negative. Total colonoscopy revealed normal mucosa without evidence of microscopic colitis or inflammatory bowel disease, on histopathology. Upper endoscopy showed normal gastroduodenal mucosa. Duodenal biopsies highlighted partial villous atrophy with severe intraepithelial lymphocytosis. Given the high suspicion of OISLE, this drug was suspended and switched to lisinopril, with replacement of electrolytes. One week after admission, the patient was discharged without diarrhea or need of nutritional/electrolyte support. Two months later, a complete recovery of weight was seen along with full normalization of laboratory tests and histopathological changes.

## 4. Discussion

CD is an immunomediated disorder occurring in people genetically susceptible to gluten and results in variable degrees of VA and an increase in intraepithelial lymphocytes [1]. The diagnosis of CD is supported by positive antibody tests (anti-tissue transglutaminase antibodies) and symptomatic and histologic response to a GFD. When celiac serologies are negative on a gluten-containing diet, alternative etiologies for VA should be considered before diagnosis of seronegative CD to prevent an unnecessary lifelong GFD [4]. Differential diagnosis includes autoimmune enteropathy,* Crohn*'s disease, microscopic enterocolitis, hypogammaglobulinemic sprue,* Whipple*'s disease, tropical sprue, intestinal tuberculosis, HIV-associated enteropathy, infectious enteritis, intestinal malignancies, and drugs-induced enteropathy. DeGaetani et al. [5] studied 72 patients with seronegative VA at a tertiary care center and, interestingly, the two most common diagnoses in these patients were seronegative CD (28%) and drug-induced sprue-like enteropathy (26%).

Olmesartan is one of the several ARBs commonly prescribed for the management of hypertension. The earliest evidence of OISLE was first described in 2012 by Rubio-Tapia et al. [6], and a few reports were published subsequently, leading Food and Drug Administration to institute label changes addressing this adverse event, in July 2013. Based on cases reported to date, this entity most frequently affects older adults (mean age, 68 years; range, 46–91 years) with no gender predominance. Symptoms of OISLE include severe chronic diarrhea (95%), weight loss (89%), fatigue (56%), nausea and vomiting (45%), abdominal pain (37%), bloating (29%), and, less commonly, reflux symptoms and loss of appetite [7]. Laboratory evaluation usually shows evidence of malabsorption with normocytic anemia, hypoalbuminemia, and multiple electrolyte abnormalities. Dehydration and acute renal failure have been reported as the main causes of hospitalization, although a case of colonic perforation has also been documented [8]. Patients can develop signs/symptoms months to years after olmesartan initiation. In the series by Rubio-Tapia et al. [6], the average duration of exposure to olmesartan was 3.1 years (range, 0.5–7 years).

Imaging and endoscopic findings in the gastrointestinal tract may reveal no significant abnormalities. Reported imaging abnormalities include diffuse edema and bowel wall thickening of the small intestine and enlarged abdominal lymph nodes. Reported abnormal endoscopic features of the small bowel include mucosal nodularity, villous atrophy, and ulceration. The most common histopathological findings are variable degrees of intestinal villous atrophy (92%). As in celiac disease, increased intraepithelial lymphocytes, according to the modified Marsh classification, are also commonly seen (61%). In addition, involvements of the stomach and colon with lymphocytic and/or collagenous gastritis and/or colitis were also reported, suggesting that this disease may affect the entire gastrointestinal tract [9]. Discontinuation of olmesartan is the mainstay of treatment. Clinical remission often occurs within a few days of stopping the drug, and almost all of the patients show histological recovery of the duodenum.

Currently, the mechanism of OISLE is unknown. The long latency period between initiation of olmesartan and development of symptoms is suggestive of cell-mediated immune damage and, furthermore, inhibitory effects of ARBs on transforming growth factor beta, an important mediator of gut homeostasis, may play a role. Rubio-Tapia et al. [6] found a prevalence of HLA-DQ2 in 68% of patients with OISLE, significantly higher than what is expected for the general population, suggesting a genetic predisposition. Scialom et al. [10] described extraintestinal autoimmune diseases in three of seven patients with OISLE. Besides this, symptomatic improvement is reported in some patients treated with steroids or other immunosuppressors prior to receiving a diagnosis of OISLE, which is supportive of an autoimmune background.

In this report, we described two uncommon cases of severe sprue-like enteropathy that achieved a remarkable improvement with suspension of olmesartan. Both patients presented with severe chronic diarrhea and significant weight loss complicated with electrolyte imbalance and/or acute renal failure. Duodenal biopsies confirmed villous atrophy and intraepithelial lymphocytosis. CD was excluded by negative conventional serologic tests and, in the first case, by the absence of clinical response to a GFD. Other causes of atrophic enteropathy were excluded and the diagnosis of OISLE was considered. Complete resolution of symptoms and laboratory changes and histological recovery after olmesartan withdrawal without any other therapeutic/dietary measures were verified, supporting the diagnosis of OISLE.

In conclusion, although OISLE is a rare cause of sprue-like enteropathy, we emphasize that this entity should be included in the differential diagnosis of seronegative villous atrophy. Considering the worldwide use of olmesartan as an antihypertensive agent, we aim to alert clinicians to this condition and other drug-induced enteropathies in patients with unexplained severe chronic diarrhea and weight loss, particularly when CD is excluded. A high index of suspicion of OISLE is important because it allows a correct early diagnosis and avoids a frustrating, unnecessary, and often expensive investigation. Up to now, a very few cases have been described and the scarcity of available data does not allow for a correct characterization of this entity, requiring additional investigations.

## References

[1] Ludvigsson J. F., Leffler D. A., Bai J. C. (2012). The Oslo definitions for coeliac disease and related terms. *Gut*.

[2] Ziegler T. R., Fernández-Estívariz C., Gu L. H., Fried M. W., Leader L. M. (2003). Severe villus atrophy and chronic malabsorption induced by azathioprine. *Gastroenterology*.

[3] Boscá M. M., Añón R., Mayordomo E. (2008). Methotrexate induced sprue-like syndrome. *World Journal of Gastroenterology*.

[4] Hall N. J., Rubin G., Charnock A. (2009). Systematic review: adherence to a gluten-free diet in adult patients with coeliac disease. *Alimentary Pharmacology and Therapeutics*.

[5] DeGaetani M., Tennyson C. A., Lebwohl B. (2013). Villous atrophy and negative celiac serology: a diagnostic and therapeutic dilemma. *The American Journal of Gastroenterology*.

[6] Rubio-Tapia A., Herman M. L., Ludvigsson J. F. (2012). Severe spruelike enteropathy associated with olmesartan. *Mayo Clinic Proceedings*.

[7] Laniro G., Bibbo S., Montalto M. (2014). Systematic review: sprue-like enteropathy associated with olmesartan. *Alimentary Pharmacology & Therapeutics*.

[8] Abdelghany M., Gonzalez L., Slater J., Begley C. (2014). Olmesartan associated sprue-like enteropathy and colon perforation. *Case Reports in Gastrointestinal Medicine*.

[9] Karen Choi E. Y., McKenn B. (2015). Olmesartan-associated enteropathy: a review of clinical and histologic findings. *Archives of Pathology & Laboratory Medicine*.

[10] Scialom S., Malamut G., Meresse B. (2015). Gastrointestinal disorder associated with olmesartan mimics autoimmune enteropathy. *PLoS ONE*.

